# The Impact of Hormonal Contraceptive Use on Serotonergic Neurotransmission and Antidepressant Treatment Response: Results From the NeuroPharm 1 Study

**DOI:** 10.3389/fendo.2022.799675

**Published:** 2022-03-11

**Authors:** Søren Vinther Larsen, Brice Ozenne, Kristin Köhler-Forsberg, Asbjørn Seenithamby Poulsen, Vibeke Høyrup Dam, Claus Svarer, Gitte Moos Knudsen, Martin Balslev Jørgensen, Vibe Gedso Frokjaer

**Affiliations:** ^1^ Neurobiology Research Unit, Copenhagen University Hospital Rigshospitalet, Copenhagen, Denmark; ^2^ Faculty of Health and Medical Sciences, University of Copenhagen, Copenhagen, Denmark; ^3^ Department of Public Health, Section of Biostatistics, University of Copenhagen, Copenhagen, Denmark; ^4^ Psychiatric Center Copenhagen, Mental Health Services in the Capital Region of Denmark, Copenhagen, Denmark

**Keywords:** hormonal contraception, oral contraception, hormonal intrauterine device, [11C]SB207145, serotonin, major depressive disorder, serotonin 4 receptor, sex steroid hormones

## Abstract

**Background:**

Hormonal contraceptive (HC) use has been associated with an increased risk of developing a depressive episode. This might be related to HC’s effect on the serotonergic brain system as suggested by recent cross-sectional data from our group, which show that healthy oral contraceptive (OC) users relative to non-users have lower cerebral serotonin 4 receptor (5-HT4R) levels. Here, we determine if cerebral 5-HT4R binding differs between HC non-users, OC users, and hormonal intrauterine device (HIUD) users among women with an untreated depressive episode. Also, we test if antidepressant drug treatment response and its association with pre-treatment 5-HT4R binding depends on HC status.

**Methods:**

[^11^C]-SB207145 Positron Emission Tomography imaging data from the NeuroPharm-NP1 Study (NCT02869035) were available from 59 depressed premenopausal women, of which 26 used OCs and 10 used HIUDs. The participants were treated with escitalopram. Treatment response was measured as the relative change in the Hamilton Depression Rating Scale 6 items (rΔHAMD_6_) from baseline to week eight. Latent variable models were used to evaluate the association between global 5-HT4R binding and OC and HIUD use as well as rΔHAMD_6_.

**Results:**

We found no evidence of a difference in global 5-HT4R binding between depressed HC users and non-users (p≥0.51). A significant crossover interaction (p=0.02) was observed between non-users and OC users in the association between baseline global 5-HT4R binding and week eight rΔHAMD_6_; OC users had 3-4% lower binding compared to non-users for every 10% percent less improvement in HAMD_6_. Within the groups, we observed a trend towards a positive association in non-users (p_adj_=0.10) and a negative association in OC users (p_adj_=0.07). We found no strong evidence of a difference in treatment response between the groups (p=0.13).

**Conclusions:**

We found no difference in 5-HT4R binding between HC users *vs*. non-users in depressed women, however, it seemed that 5-HT4R settings differed qualitatively in their relation to antidepressant drug treatment response between OC users and non-users. From this we speculate that depressed OC users constitutes a special serotonin subtype of depression, which might have implications for antidepressant drug treatment response.

## Introduction

Hormonal contraception (HC) is used by millions of women worldwide to avoid pregnancies as well as for other indications such as dysmenorrhea, menorrhagia, and acne (1, 2). In spite of HC being used for more than 60 years it is still debated whether HC use causes mood deterioration and development of depressive episodes (3). Recent findings from large epidemiological studies have suggested that starting on HC is associated with an increased risk of being diagnosed with Major Depressive Disorder (MDD), being prescribed antidepressants (4, 5), and even attempting or completing suicide (6–8). Notably, the risk is higher when HC is initiated in adolescence (9, 10) and moreover, HC use in adolescence is associated with lasting vulnerability for MDD in adulthood (11), which may relate to HC being introduced in a critical stage of brain development (12). Even though HCs are widely used, we still have limited understanding on how HC affects the brain and which implications it may have for mental health (13).

HCs exist in different types in terms of hormonal content and route of administration such as oral contraceptives (OCs), hormonal intrauterine devices (HIUDs), vaginal rings, injections and subdermal implants (14). OCs and HIUDs are the types most widely used (1). HCs contain synthetic female sex hormones either in form of a progestin alone (HIUDs and progesterone-only pills) or in a combination with an estrogen (combined OCs), most frequently ethinylestradiol (14). The synthetic steroids in combined OCs and in high-dose progesterone-only pills act by suppressing the hypothalamic-pituitary-gonadal hormonal axis resulting in suppression of the endogenous hormone production, disrupted follicular maturation, and inhibition of ovulation (15). HIUDs and low-dose progesterone-only pills on the other hand only inhibit ovulation in 60-85% of the time and even as low as 15% of the time after one year of HIUD use. Instead, their primary mechanism of action is induction of local inflammation in the cervical mucous to prevent access of sperm and by thinning of the uterus lining to prevent implantation of a fertilized egg (16, 17). Further, the synthetic steroids in HC induce an increase in the sex hormone binding globulin level further lowering the bioavailable fraction of sex hormones (18). These profound changes in the sex hormone milieu may shape brain biology both in terms of brain structure (19) and function (20). Recently, we have shown that OC use appears to affect the brain’s serotonin signaling system (21), which is a key system for maintaining mental health and is involved in the pathophysiology of MDD and the treatment hereof (22). We used the molecular imaging technique ([^11^C]SB207145-radioligand positron emission tomography (PET)) to quantify the postsynaptic serotonin 4 receptors (5-HT4R). The 5-HT4Rs are sensitive to chronic synaptic serotonin manipulation such that they are inversely correlated to serotonergic tonus (23–25) making it an interesting tool to study MDD pathophysiology. In a population of healthy women, we found that women using OCs had 9-12% lower 5-HT4R level globally in the brain compared to non-users (21). In comparison, our group also found 7-8% lower 5-HT4R global binding in unmedicated depressed individuals compared to healthy controls, and intriguingly, this gap was only evident in those who remitted after eight weeks of antidepressant treatment with a selective reuptake inhibitor (SSRI), indicating that those responding to the treatment may have a serotonergic subtype of MDD (26). The SSRI works by targeting the serotonin system, which induces an additional downregulation of 5-HT4R levels in neostriatum, a phenomenon confirmed in both a depressed and healthy cohort (23, 26). From this, we speculate whether the mechanisms causing the lower 5-HT4R binding in OC users and depressed individuals are similar and if it has any implications for the SSRI treatment response in women with a depressive episode.

We sought to investigate this by determining 1) if the 5-HT4R binding differs between depressed women who do not use HC *vs*. those using OCs and HIUDs, 2) if antidepressant drug treatment response is affected by HC use, 3) if an association between pre-treatment 5-HT4R brain binding and antidepressant drug treatment response depends on HC status, and 4) if neostriatal 5-HT4R levels are sensitive to SSRI treatment in HC users. We hypothesize that if the effects from OC and MDD are not additive, we will not be able to detect a difference in baseline 5-HT4R binding. However, when we account for treatment response, and thus may account for the serotonergic subtype of MDD, we will be able to detect a negative main effect of OC use on 5-HT4R binding. In line with that, we hypothesize that an association between baseline binding and treatment response will depend on OC use, such that an association is found in non-users but not in OC users, as they may constitute a more homogenous group in terms of 5-HT4R downregulation. We further hypothesize that the effect of HIUD use on 5-HT4R binding will be in the same direction as of OC use, but with smaller effect size, due to the smaller degree of synthetic steroid exposure and larger degree of preserved ovulations (i.e., preserved hormonal cycle).

## Methods

We included patient data from the NeuroPharm-NP1 clinical trial (27), which aimed to predict MDD treatment outcome with the use of potential biomarkers including 5-HT4R PET brain scans. The study was approved by the Committees on Health Research Ethics in the Capital Region of Denmark (H-15017713), the Danish Data Protection Agency (04711/RH-2016-163), and the Danish Medicines Agency (NeuroPharm-NP1, EudraCT-number 2016-001626-34) and was pre-registered on clinicaltrials.gov (NCT02869035). A detailed description of the study design and the study elements can be found in the clinical trial protocol (27). The present study used information on HC use and baseline 5-HT4R PET imaging data together with clinical outcome measures after eight weeks of antidepressant drug treatment. It represents initially unplanned analyses motivated by the observed difference in 5-HT4R binding in healthy women who use OC ***vs***. those who do not use OC (21). The treatment started at a daily dose of 10 mg of the SSRI, escitalopram, and was increased to a daily dose of maximally 20 mg, depending on treatment response and adverse reactions. In case of non-response at week four, patients were switched to duloxetine, a serotonin-norepinephrine reuptake inhibitor (SNRI). Compliance was confirmed by serum drug levels after eight weeks of treatment. A subset of the study population was rescanned after eight weeks of treatment to map the change in 5-HT4R level. Allocation to rescan happened continuously until allotted rescans were completed.

### Study Population

The study population consists of all the depressed premenopausal women (defined as < 50 years of age) from the NeuroPharm-NP1 study with available baseline 5-HT4R PET data and information on HC use. One patient was excluded from the analyses on 5-HT4R imaging data due to an interrupted PET scan (n=59 for the first analysis). Eight patients dropped out during follow-up, six OC users and two non-users (n=52 for the second and n=51 for the third analysis). Twenty-six of the remaining women were rescanned at follow-up; 15 were non-users, six were OC users and five were HIUD users (n=26 for the fourth analysis). The women were suffering from an unmedicated moderate to severe (> 17 points on the Hamilton Depression Rating Scale 17 items (HAMD_17_)) depressive episode confirmed by a psychiatrist at inclusion. The current depressive episode was single or recurrent and did not involve acute severe suicidal ideation or psychosis. They had no prior or present history of other major psychiatric disorders confirmed by use of the diagnostic tool, the Mini International Neuropsychiatric Interview version 6.0 (28), or any other severe somatic illness confirmed by a basic somatic screening. Level of education was calculated as the number of completed school years (7-12 years) summed with a Likert score indexing highest completed or commenced degree ranging from one (no vocational degree) to five (>four years of higher learning at university level). Information about relationship status was acquired *via* interview. Blood tests to assess plasma estradiol and progesterone levels were taken at the baseline PET scan as part of standard biochemical screening.

### Hormonal Contraception

Information about HC use was acquired *via* face-to-face interview at the time of the PET scan and *via* a written questionnaire which included a question on the specific HC type. The HC type was divided into HIUD and OC. OCs included different generations of combined OCs (n=20) and progestogen-only pills (n=6), as specified in **Table S1**.

### Clinical Outcome Measure

The clinical outcome measure was the relative change in Hamilton Depression Rating Scale 6 items (rΔHAMD_6_) in percentage from baseline to week eight. In the larger NeuroPharm-NP1 study, the categorical treatment response categories, remitters *vs*. non-responders, were used as the primary clinical outcome and rΔHAMD_6_ was included as the secondary outcome (27). For this study, which only includes the women, we only applied rΔHAMD_6_ to avoid underpowered statistics.

### Imaging

PET acquisition and quantification is detailed in the trial protocol (27) and briefly summarized here: A high-resolution research tomography Siemens PET scanner (CTI/Siemens, Knoxville, TN, USA) (256 × 256 × 207 voxels; 1.22 × 1.22 × 1.22 mm) was used for acquiring PET scans. Each scan was obtained from a 120 min dynamic PET acquisition immediately after a 20 second intravenous bolus injection of the [^11^C]SB207145 tracer ligand. Motion correction was performed using the AIR 5.2.5 software (29). High-resolution structural T1-weighted magnetic resonance images were acquired on a Siemens 3-Tesla Prisma scanner with a 64-channel head coil. The images were segmented into cerebrospinal fluid, white- and gray matter and were co-registered with PET images using the Statistical Parametric Mapping software (SPM8, The Wellcome Centre for Human Neuroimaging, UCL, London, UK). Regions were automatically delineated from the MR image *via* the user-independent algorithm in the Pvelab software (30). Regions of interest (ROIs) were neostriatum, hippocampus and neocortex, as these were lower in 5-HT4R binding in the depressed cohort in the NeuroPharm-NP1 Study (26), and as these regions represent low, intermediate, and high expression levels of brain 5-HT4Rs (31) and lastly, as these regions have previously shown lower 5-HT4R levels in OC users (21). As previous studies only found an effect of SSRI treatment on 5-HT4R binding in neostriatum, we only used this region to test whether this was also true in the HC users. Regional non-displaceable binding potentials (BP_ND_) were quantified using the simplified reference tissue model with cerebellum as reference tissue (32).

### Statistics

For the descriptive statistics, we compared between the HC non-users, the OC users, and the HIUD users, and p-values were computed with Fisher’s exact test for the categorical outcomes, Kruskal-Wallis Rank Sum Test for the continuous and the discrete variables and when relevant, Dunn’s test was used for *post hoc* analyses where multiple comparisons were corrected for with the Bonferroni-Holm method. For plasma progesterone and estradiol, we used Gehan test (33) due to censored values (18 estradiol- and 35 progesterone samples were below the detection limit of 0.09 nM and 0.6 nM, respectively).

To evaluate if HC use was associated with global 5-HT4R brain binding (aim 1), we used a linear latent variable model (LVM) where the effects of HIUD- and OC use were mediated through a shared latent variable (hereafter phrased as the global LV) across brain regions of low (neocortex), intermediate (hippocampus) and high (neostriatum) 5-HT4R binding (31). By introducing the global LV, we were able to account for the large inter-correlation in 5-HT4R binding between brain regions. The binding in each region was adjusted independently, i.e., not through the global LV, for age, BMI, 5-HTTLPR genotype (LALA or non-LALA), and injected [11C]SB207145 mass per kg bodyweight as these are considered to influence 5-HT4R PET measurements (34–36). 5-HT4R BP_ND_ values were log-transformed prior to modeling and the regional estimates are expressed as a percent difference in 5-HT4R binding in the OC users *vs*. the non-users and in the HIUD users *vs*. the non-users. To test if the treatment response differed between the groups (aim 2), we used the Kruskal-Wallis Rank Sum Test. For reporting the effect size, we used the Mann-Whitney parameter (37), which gives the probability that a randomly selected individual from one group had a worse treatment response compared to one from another group. An estimate of 0.5 indicates no difference in treatment response. To evaluate if an association between baseline 5-HT4R BP_ND_ and week eight rΔHAMD_6_ depends on OC/HIUD use (aim 3), we extended our LVM by including an interaction term between rΔHAMD_6_ and the HC group to be mediated through the global LV. The estimates related to rΔHAMD_6_ are expressed as the percent change in 5-HT4R BP_ND_ per 10% change in HAMD_6_ (r_10_ΔHAMD_6_). To evaluate the effect of eight weeks of SSRI/SNRI treatment on neostriatal 5-HT4R binding across groups (aim 4), we used linear regression adjusting for the difference in injected [11C]SB207145 mass per kg bodyweight between baseline and week eight scans. Due to the smaller re-test samples within the OC and the HIUD group, the p-values were also computed with a permutation test with 10,000 permutations.

Diagnostic tools were used to assess the adequacy of the models’ assumptions. We used chi-squared tests to evaluate specification of the covariance structure for each LVM with chi-square < 0.05 indicating suboptimal specification [section 6.2.4 in (38)]. Missing data in the analyses regarding treatment response were handled using complete case analysis. Two-sided statistical tests were used and p < 0.05 was considered statistically significant. Confidence intervals and p-values computed for the regional effects were adjusted for three comparisons using the single-step Dunnett’s procedure (39). Statistical analyses were performed in R (40) and LVMs were estimated with the lava package (38).

## Results

### Study Population Profile

The clinical profile and PET parameters of the study population at baseline are shown in **Table 1**. The non-users, the HIUD users, and the OC users were similar in terms of proportion suffering from first depressive episode and in clinician rated depressive symptoms (HAMD_6/17_) at baseline. The depressive symptoms scored within a moderate-to-severe depressive episode as restricted by the inclusion criteria. The educational level tended to differ between the groups (p=0.06) such that the HC users tended to have lower education. As expected, the OC users had lower plasma estradiol levels (median [Q1, Q3]: 0.09 nM [0.09, 0.18]) compared to the non-users (median [Q1, Q3]: 0.30 nM [0.16, 0.64]), p_adj_=0.0002, and the HIUD users (median [Q1, Q3]: 0.23 [0.17, 0.26]), p_adj_=0.01. The OC users had lower plasma progesterone levels but only compared to the non-users (p_adj_=0.04). The HIUD users did not seem to differ from the non-users in plasma hormone levels.

**Table 1:** Clinical profile and PET parameters at baseline.

	Non-users (n=23)	HIUD users (n=11)	OC users (n=26)		
	n (%)	n (%)	n (%)	p-value^α^	n
First MDD episode	10 (43.5%)	5 (45.5%)	8 (30.8%)	0.61	60
5-HTTLPR L_A_L_A_ genotype	8 (34.8%)	1 (9.1%)	7 (26.9%)	0.32	60
In relationship	7 (30.4%)	6 (54.5%)	9 (34.6%)	0.47	60
	**Median [Q1, Q3]**	**Median [Q1, Q3]**	**Median [Q1, Q3]**	**p-value** ^β^	**n**
Age	26.2 [22.9, 30.3]	23.7 [22.5, 24.4]	23.3 [21.6, 25.7]	0.25	60
BMI [kg/m^2^]	23.9 [20.5, 31.2]	22.1 [19.2, 23.9]	21.8 [20.0, 24.7]	0.31	60
Educational level	16.0 [16.0, 17.0]	15.0 [12.5, 16.5]	14.5 [13.0, 16.0]	0.06	54
HAMD_6_	12.0 [11.5, 13.5]	13.0 [12.0, 13.5]	12.5 [12.0, 13.0]	0.87	60
HAMD_17_	22.0 [21.0, 24.0]	25.0 [21.0, 27.0]	23.0 [20.2, 25.0]	0.58	60
P-estradiol [nM]	0.30 [0.16, 0.64]	0.23 [0.17, 0.26]	0.09 [0.09, 0.18]	0.20^γ^	60
				0.0002^δ^	
				0.01^ϵ^	
P-progesterone [nM]	0.90 [0.60, 15.00]	0.60 [0.60, 3.85]	0.60 [0.60, 0.67]	0.59^γ^	60
				0.04^δ^	
				0.59^ϵ^	
Injected dose [MBq]	600.2 [573.6, 604.2]	591.3 [526.8, 602.4]	602.5 [589.8, 605.2]	0.21	59
Injected tracer mass/kg [µg/kg]	8.6x10^-3^ [6.5x10^-3^, 1.3x10^-2^]	8.6x10^-3^ [6.0x10^-3^, 1.7x10^-2^]	9.2x10^-3^ [6.4x10^-3^, 1.2x10^-2^]	0.91	59
Cerebellum, area under curve [kBq/ml]	10380.8 [9105.6, 12660.3]	10755.5 [9055.6, 11657.2]	11118.2 [9058.6, 13413.4]	0.47	59

HIUD, hormonal intrauterine device; OC, oral contraceptive; MDD, Major Depressive Disorder; BMI, Body Mass Index; HAMD_6/17_, Hamilton Depression Rating Scale 17 or 6 items; MDI, Major Depressive Inventory; ^α^p-values are computed with Fischer’s exact test. ^β^p-values are computed with Kruskal-Wallis Rank Sum Test except for P-estradiol and P-progesterone, for which Gehan test was used due to censored values and they were corrected for three comparisons with the Bonferroni-Holm method. ^γ^Non-users vs. HIUD users. ^δ^Non-users vs. OC users. ^ϵ^HIUD users vs. OC users.

### Hormonal Contraception and Serotonin 4 Receptor Binding

**Figure 1:**
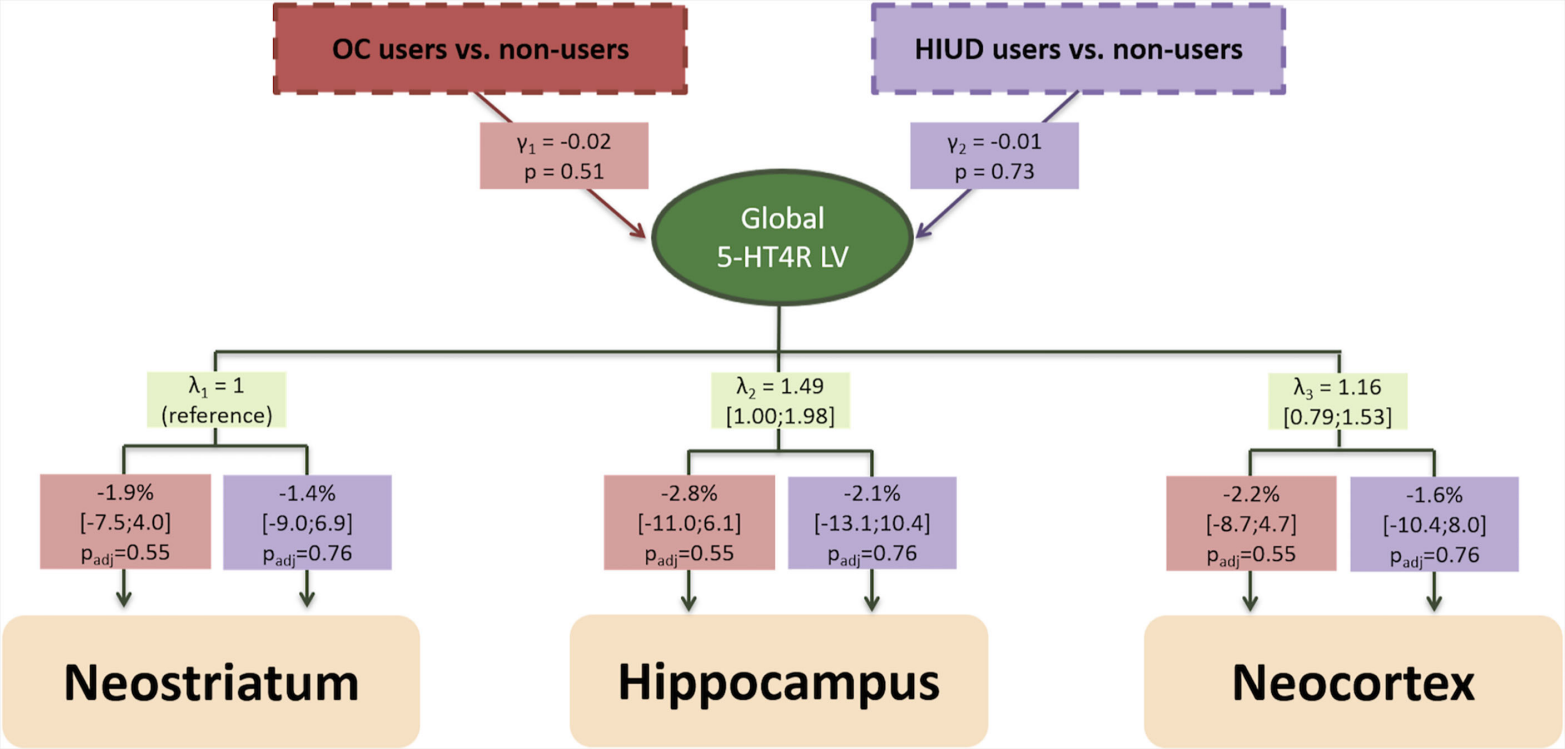
The estimated latent variable model for the effect of oral contraceptive (OC) and hormonal intrauterine device (HIUD) use on baseline 5-HT4R BP_ND_ in women with an untreated depressive episode. γ is the effect on the global latent variable interpreted as global (log-transformed) 5-HT4R BP_ND_ effects. λ is the loading on each region. The boxes beneath the loadings indicate the percentage difference in 5-HT4R binding for each brain region in OC- and HIUD users compared to non-users. Regional bindings were adjusted for Age, BMI, 5-HTTLPR genotype and injected tracer mass per kg bodyweight (not shown). P-values and confidence intervals are adjusted for 3 comparisons by use of the Dunnett’s test.

In support of the LVM structure, we found significant region-specific bindings loading onto the global LV for all regions (p<0.001). From the chi-squared test we found no evidence for a lack of fit (p_adj_>0.79), so no additional covariance was added to the model. There was no evidence for an association between 5-HT4R binding and OC use (p=0.51) or HIUD use (p=0.73) (**Figure 1**). The corresponding non-significant regional estimates for OC use varied between -2.8% and -1.9%, and for HIUD use between -2.1% and -1.4%. No gross deviation from the normality assumption was observed.

### Antidepressant Treatment Response

**Figure 2:**
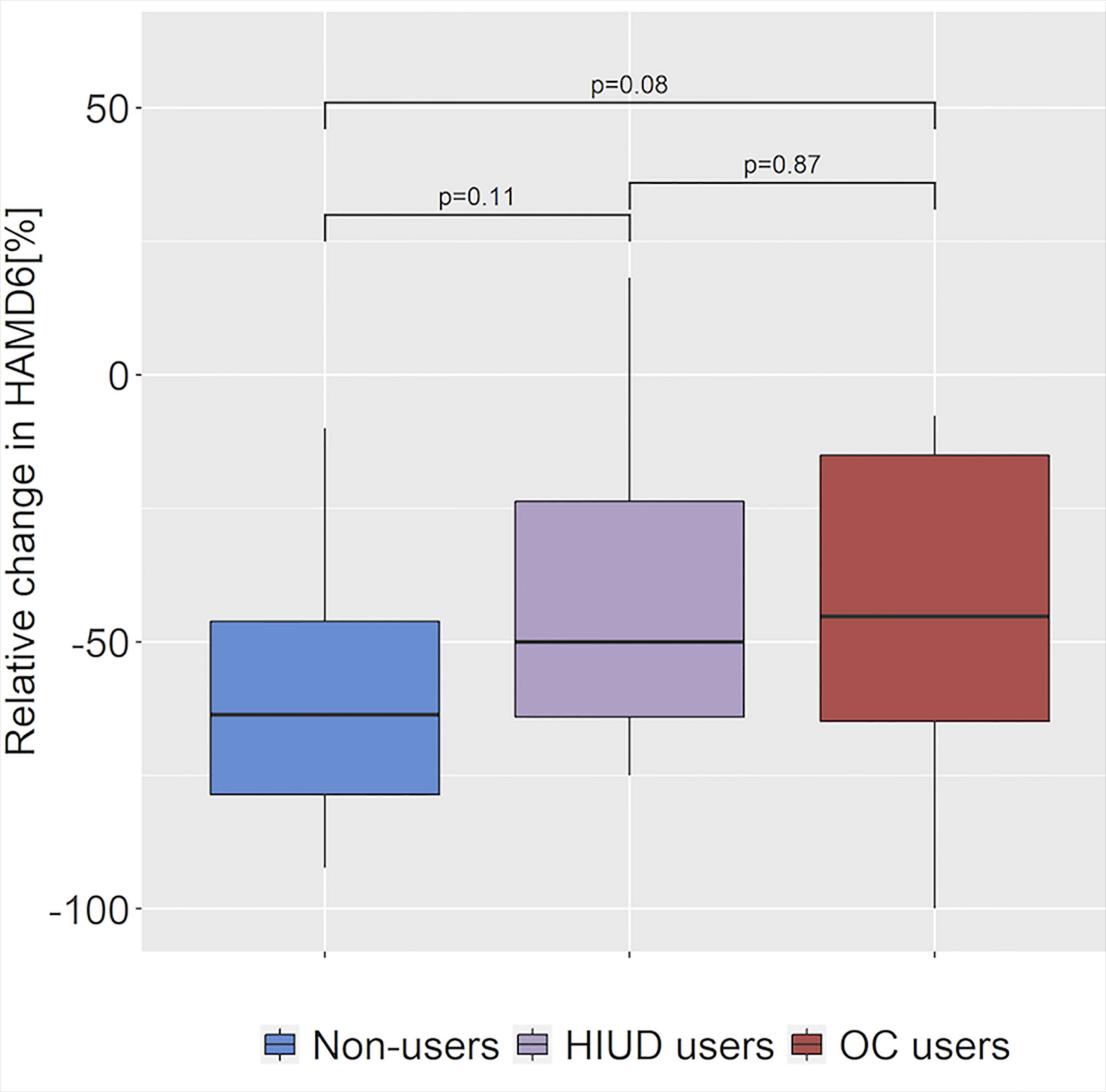
Antidepressant drug treatment response at week eight across hormonal contraceptive user status. Difference in relative change in Hamilton Depression Rating Scale 6 items (HAMD6) from baseline between the HC non-users, the hormonal intrauterine device (HIUD) users and the oral contraceptive (OC) users. A larger negative change corresponds to a better improvement of depressive symptoms. P-values are computed with Mann-Whitney tests with no correction for multiple comparisons.

Week eight depression profiles are shown in **Table 2**. We found no evidence of a difference in proportion of patients who switched to duloxetine after week four (p=0.34) or in week eight treatment response (rΔHAMD_6_) between the groups (p=0.13).The estimated probability of finding a smaller reduction in HAMD_6_ in an OC and an HIUD user was 66% (95% CI: [0.48; 0.80], p=0.08) and 68% (95% CI: [0.46; 0.83], p=0.11), respectively, compared to in a non-user (**Figure 2**), and notably, this was at a non-significant level as the confidence intervals included 0.5 (p-values were not corrected for multiple comparisons). The OC users seemed to require higher doses of escitalopram compared to the non-users (p_adj_=0.04) and the HIUD users (p_adj_=0.01); 77.8% of the OC users were treated with the highest recommended daily dose of 20 mg compared to 35.0% and 22.2% of the non-users and the HIUD users, respectively. Correspondingly, the OC users also had a higher plasma concentration of escitalopram at week eight compared to the HIUD users (p_adj_=0.03), and at a trend level compared to the non-users (p_adj_=0.06). The dropouts’ depression profiles, in terms of baseline HAMD_6/17_ as well as relative change in HAMD_6_ in percentage from baseline to week one, two, four and eight and reason for dropout, are presented in **Table S2**.

**Table 2:** Clinical depression profile at week eight.

	Non-users (n=21)	HIUD users (n=11)	OC users (n=20)		
	**n (%)**	**n (%)**	**n (%)**	**p-value^α^**	**n**
Switchers to duloxetine	1 (4.8%)	2 (18.2)	2 (10)	0.34	52
	**Median [Q1, Q3]**	**Median [Q1, Q3]**	**Median [Q1, Q3]**	**p-value** ^β^	**n**
rΔHAMD_6_ [%]	-63.6 [-78.6, -46.2]	-50.0 [-64.1, -23.7]	-45.3 [-64.9, -15.1]	0.13	52
P-escitalopram [nM]	68.1 [43.3, 102.8]	42.5 [36.0, 108.5]	105.7 [74.1, 139.8]	0.40^γ^	47
				0.06^δ^	
				0.03^ϵ^	
	**n (%)**	**n (%)**	**n (%)**	**p-value^α^**	**n**
Escitalopram dose:					
5 mg	0 (0.0)	1 (11.1)	0 (0.0)	0.26^γ^	47
10 mg	2 (10.0)	2 (22.2)	1 (5.6)	0.04^δ^	
15 mg	11 (55.0)	4 (44.4)	3 (16.7)	0.01^ϵ^	
20 mg	7 (35.0)	2 (22.2)	14 (77.8)		

HIUD, hormonal intrauterine device; OC, oral contraceptive; rΔHAMD_6_, relative change in Hamilton Depression Rating Scale 6 items from baseline. ^α^p-value is computed with Fischer’s exact test. ^β^p-values are computed with Kruskal-Wallis Rank Sum Test and, for post hoc analyses, with Dunn’s test corrected for three comparisons with the Bonferroni-Holm method. ^γ^Non-users vs. HIUD users. ^δ^Non-users vs. OC users. ^ϵ^HIUD users vs. OC users.

### Pre-treatment Serotonin 4 Receptor Binding and Antidepressant Treatment Response

**Figure 3:**
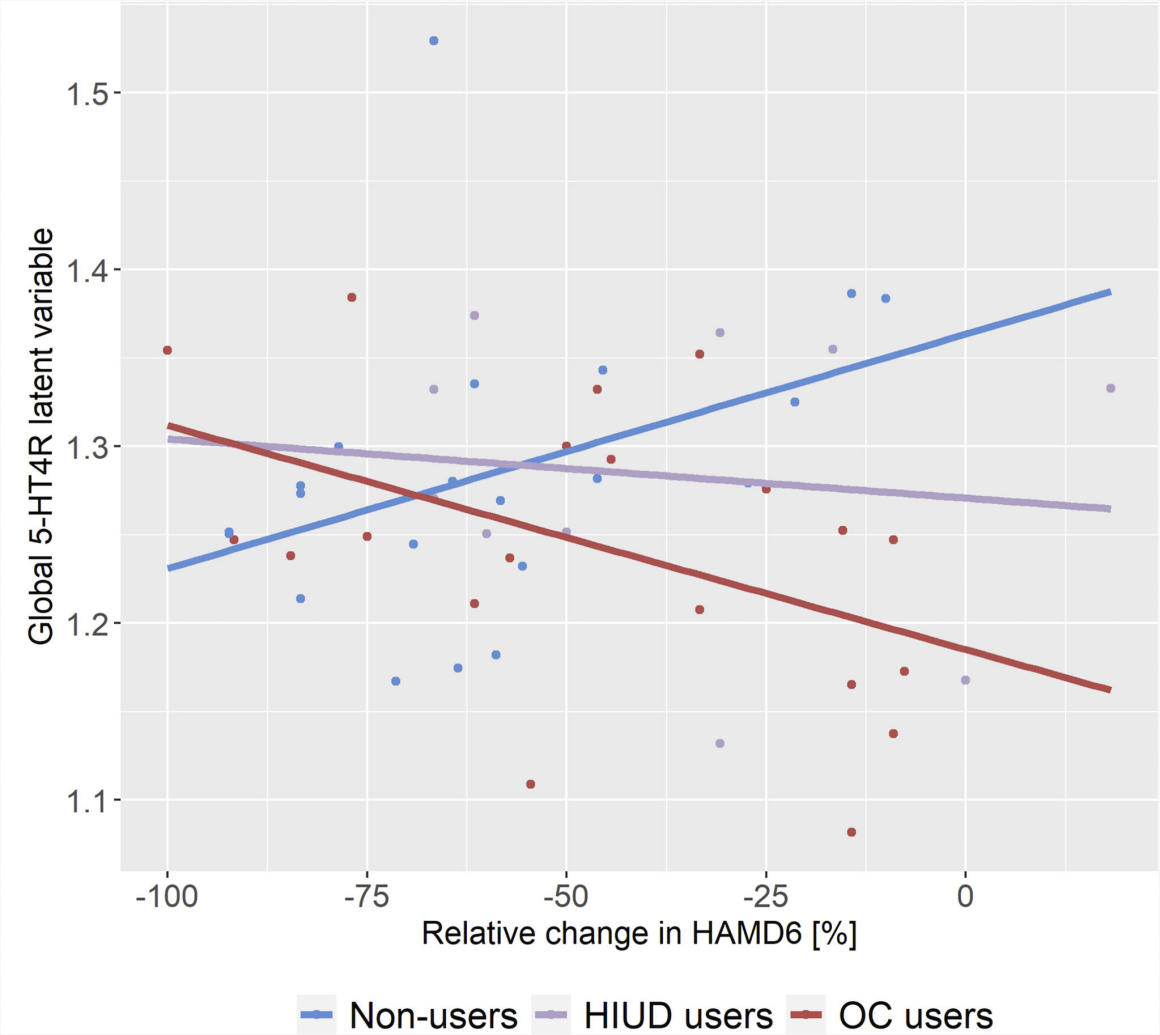
Estimated global 5-HT4R latent variable summarizing the association between baseline global 5-HT4R brain binding and week eight antidepressant drug treatment response across the groups. The slopes are the association between baseline (un-medicated) 5-HT4R binding and change in Hamilton Depression Rating Scale 6 items (HAMD_6_) in each group, respectively. A negative change in HAMD_6_ mirrors an improvement of depressive symptoms. The slopes are 1.33x10^-2^ (p_adj_=0.10) for the non-users, -3.34x10^-3^ (p_adj_=0.75) for the hormonal intrauterine device (HIUD) users, and -1.27x10^-2^ (p_adj_=0.07) for the oral contraceptive (OC) users.

**Figure 4:**
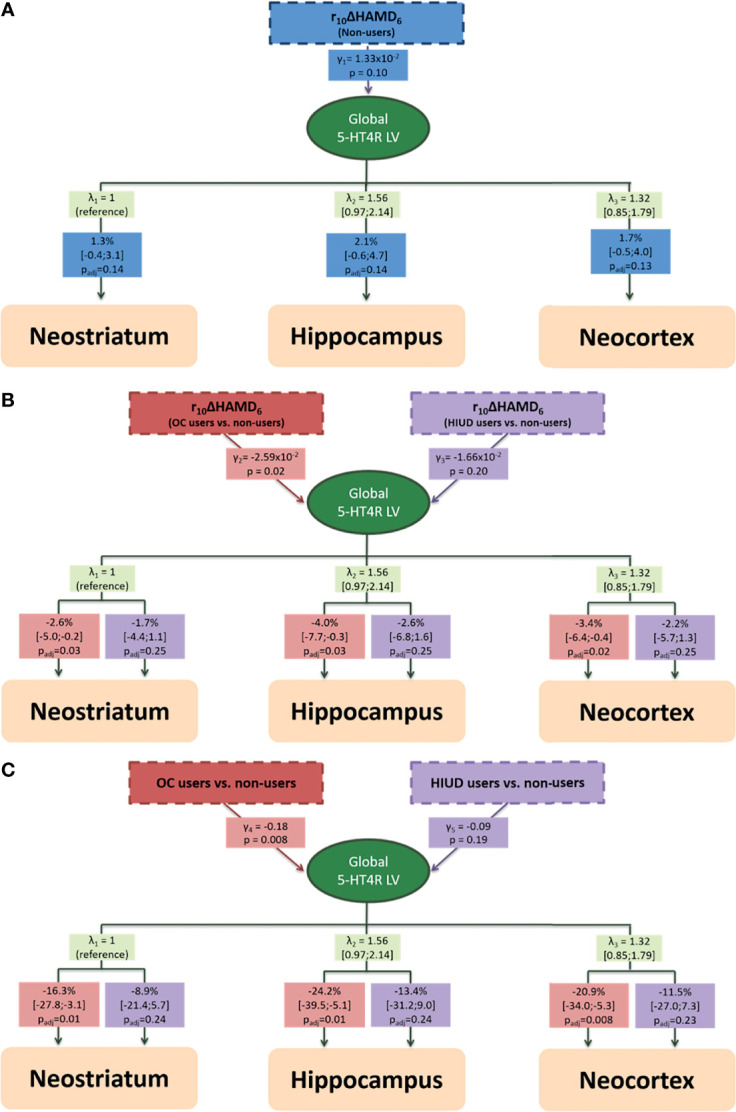
The estimated latent variable model for the association between baseline 5-HT4R binding and week eight antidepressant treatment response as a function of hormonal contraceptive status shown in three layers **(A–C)**. **(A)** The association between baseline 5-HT4R binding and treatment response in non-users. **(B)** The difference in the association between baseline 5-HT4R binding and treatment response between the non-users and the oral contraceptive (OC) and the hormonal intrauterine device (HIUD) users, respectively. **(C)** The estimated effect of OC and HIUD use on 5-HT4R binding when no change in Hamilton Depression Rating Scale 6 items (HAMD6). γ is the estimates of the association with the global latent variable interpreted as global (log-transformed) 5-HT4R BP_ND_ estimates. λ is the loading on each region. The boxes beneath the loadings indicate the percentage difference in 5-HT4R binding for each brain region. Regional bindings were adjusted for Age, BMI, 5-HTTLPR genotype and injected tracer mass per kg bodyweight (not shown). P-values and confidence intervals are adjusted for 3 comparisons. r_10_ΔHAMD_6_: 10% relative change in Hamilton Depression Rating Scale 6 items.

Again, the LVM was supported by the region-specific bindings loading onto the global LV (p<0.001). The chi-squared test showed no evidence of misspecification of the covariance structure (p_adj_>0.41). The estimated associations between baseline global 5-HT4R binding (represented as the global LV) and treatment responses across each group are shown in **Figure 3** with a trend towards a positive slope in the non-users (1.33x10^-2^ (p=0.10)), a trend towards a negative slope in the OC users (-1.27x10^-2^ (p=0.07)), and a non-significant negative slope in the HIUD users (-3.34x10^-4^ (p=0.75)). The corresponding correlation coefficients are 0.39 for the non-users, -0.46 for the OC users, and -0.12 for the HIUD users. In the OC users, the slope differed by -2.59x10^-2^ (p=0.02) from the non-users. In **Figure 4**, the LVM is summarized in three layers (A, B and C). The first (A) shows the trend towards a positive association between the 5-HT4R binding and r_10_ΔHAMD_6_ mediated through the global LV in the non-users (p=0.10). The second layer (B) shows how this association differs in the HC users from the non-users. The regional estimates show that per 10% less improvement in HAMD_6_, the OC users had 2.6-4.0% (p_adj_ ≤ 0.03) lower binding at baseline compared to the non-users. The third layer (C) shows the estimated effect of HC use on 5-HT4R binding if no treatment response was achieved at week eight, i.e., the estimated HC effect when accounting for the confounding factor of a suggested serotonergic subtype of MDD. However, as we have only few observations at this part of the response scale, these estimates are calculated based on the model and less on actual observed differences. At the regional level, the percent effect of OC use varied between -24.2% and -16.3% (p_adj_ ≤ 0.01). No gross deviation from the normality assumption was observed for any of the LVMs.

### Antidepressants' Effect on Serotonin 4 Receptor Binding

**Figure 5:**
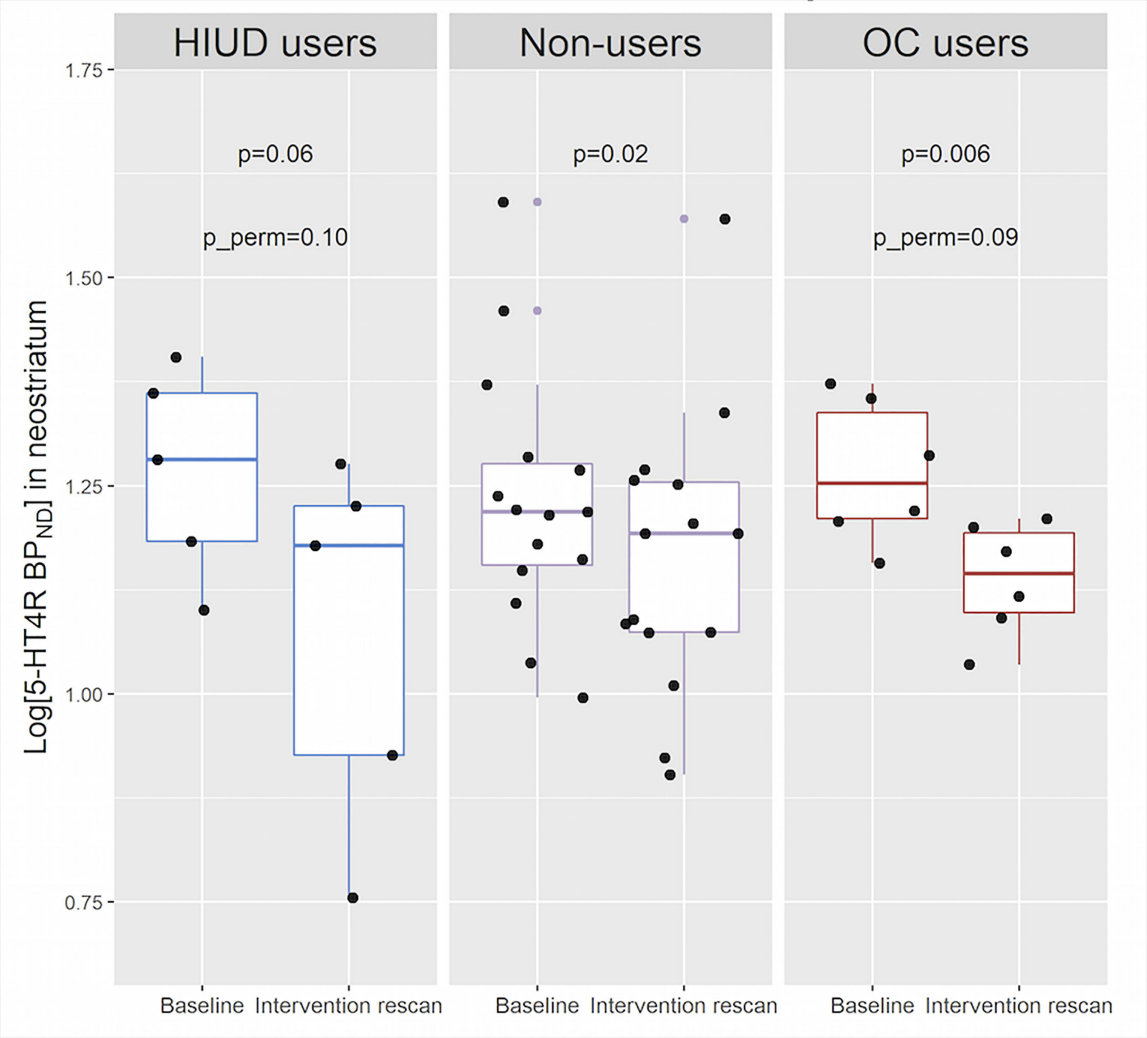
The effect of eight weeks of SSRI/SNRI treatment on neostriatal 5-HT4R binding across the groups. p_perm: p-values based on 10,000 permutations to account for the small sample sizes in the oral contractive (OC)- and the hormonal intrauterine device (HIUD) user groups.

In **Figure 5**, we show the effects of eight weeks of SSRI/SNRI treatment on the 5-HT4R BP_ND_ across the groups. A reduction in 5-HT4R BP_ND_ appeared to be present in all groups with an estimated reduction in the non-users of 7.0% (95% CI: [-12.1; -1.6], p=0.02). Due to the small sample sizes in the HIUD- and the OC group, the effect sizes are less reliable, and the p-values are also computed after 10,000 permutations. It revealed that, if the null hypothesis is true (i.e., no change in 5-HT4R BP_ND_ after eight weeks of SSRI treatment), the observed lower 5-HT4R BP_ND_ would be found by chance in 9-10% of the cases, hence, a reduction in 5-HT4R binding is only found at a trend level in the HIUD and the OC users.

## Discussion

In this study, we found no evidence of a difference in 5-HT4R binding in unmedicated depressed women who use OCs or HIUDs relative to non-users. We found a trend towards an association between baseline global 5-HT4R BP_ND_ and treatment response in the non-users and the OC users and these were in opposite directions with a significant crossover interaction, such that the OC users had 3-4% lower binding compared to the non-users for every 10% percent less improvement in HAMD_6_ at week eight. Based on our model, the main effect of OC use on regional 5-HT4R binding at zero improvement in depressive symptoms was estimated to be between -16 and -24%. In addition, we observed no strong evidence of a difference in treatment response between the groups, although, the estimated probability of finding a smaller reduction in HAMD_6_ in an OC and an HIUD user was numerically higher compared to in a non-user. Last, we found that as in the non-users, both the HIUD users and the OC users seemed to react to SSRI treatment in terms of neostriatal 5-HT4R downregulation, however, only at a trend level.

### Hormonal Contraceptive Use and Brain Serotonin

In a healthy population we found OC use to be associated with 9-12% lower global 5-HT4R binding (21), which was not seen in this depressed cohort, however, the depressed cohort is also 7-8% lower in binding compared to a healthy population (26). Hence, the OC effect on 5-HT4R binding may be obscured by the effects of the MDD as expected, and thus we see no indication of the effects of OC and MDD being additive. Comparable to OC use, we found a numerically lower, but still statistically non-significant effect of HIUD use. In the previous study on the healthy population (21), we were underpowered to investigate an effect of HIUD use. Thus, brain signatures of HIUD use remain to be studied since HIUD use is also associated with an increased risk of developing MDD (4, 5).

Whereas we found no difference in 5-HT4R binding level at baseline between the groups, we observed a crossover interaction between the OC users and the non-users in the association between baseline 5-HT4R BP_ND_ and treatment response, but notably, the association within each group was only borderline significant, which may be due to power issues. As we were not expecting to see a negative trend in the association in the OC users, this highlights the question whether the 5-HT4R setting may differ qualitatively, i.e., whether the mechanisms behind a lower 5-HT4R level differ. As shown in a mixed male-female cohort of MDD patients, only those responding to treatment had a lower baseline (unmedicated) 5-HT4R level compared to healthy controls, so it has been speculated whether they have a serotonergic subtype of MDD, in which the lower binding is due to a compensatory upregulation of the serotonergic tonus as an attempt to maintain euthymia (26). In contrast, we speculate whether all depressed OC users belong to a special serotonergic subgroup with varying degree of serotonergic involvement. We suggest that the mechanism affecting the 5-HT4R level in OC users may be driven by a hormone-dependent decrease in gene expression levels due to a suppressed hormone state in OC users, which align with previous observations in healthy OC users (21). This also aligns with preclinical research showing that estradiol supports serotonergic signaling in terms of increased capacity of serotonin synthesis and reuptake (41, 42), reduced capacity of serotonin degradation (41), increased neural firing (43) and increased serotonin receptor availability (44), which mainly happen *via* gene expression through estrogen receptor alpha and -beta (45). Also notably, estradiol increases 5-HT4R mRNA expression in the anterior pituitary cells in rats (46). We speculate that the more the 5-HT4R expression level as well as other parts of the serotonin signaling are compromised by the suppressed hormone state in OC users, the worse the treatment response, and in contrast, only the non-users with a serotonergic component show a better response to an SSRI targeting this system. If this theory is applied on our results and we compare the 5-HT4R levels at rΔHAMD_6_ equal to 0% (i.e., the results from the third layer of the LVM), we compare the effect of OC use with a population with a non-serotonergic subtype of MDD, which reveals an OC effect equal to about -20%. However, since this result is based on our model with rather few observations in this range of the treatment response, it should be interpreted with some caution.

The association between baseline binding and treatment response did not differ between the HIUD users and the non-users, however, the estimate was in the same direction as in the OC users. It is possible that, if replicated in larger sample sizes, this reflects an effect of HIUD use on serotonin brain signaling.

### Hormonal Contraceptive Use and Antidepressant Treatment Response

The estimated probability of finding a smaller reduction in HAMD_6_ in an OC and an HIUD user compared to a non-user was 66% and 68%, respectively, but the confidence intervals included 50%, so we have insufficient evidence to conclude that differences in the treatment response exist. This might be a question of power as the confidence intervals were wide, e.g., the OC users *vs*. the non-users 95% confidence spanned from 48% to 80%. Larger samples would help narrow the confidence intervals to help us discriminate between differences in treatment response *vs*. no or very small differences. The median rΔHAMD_6_ was, at the non-significant level, about 18 percent point and 14 percent point lower in the OC and the HIUD users, respectively, relative to the non-users. This is equivalent to about two points based on median HAMD_6_ scores at baseline. Treatment response could be affected by educational level (47) as the non-users tended to have higher educational level, however, this could also be related to the HC users not having started their final degree, yet, as the median age in the HC users was about 23 *vs*. 26 in non-users. Adjusting for educational level did not have any notable impact on the result (not shown). Limited and insufficient clinical evidence exists addressing whether HC use affects SSRI treatment response. The STAR*D study reported a trend towards better remission rates to citalopram treatment in 226 HC users compared to 670 HC non-users, however, this effect was not robust to adjustment for potential confounders (48). Another study, based on 17 double-blind, placebo-controlled clinical trials with 1698 women, found no difference in treatment response between the OC users and the non-users measured by change in HAMD_17_ (49). It has been highlighted that the studies are limited by lacking information on the types of HCs used, for not including or missing response rates across the trials, and for including studies with variable SSRI dosages (50). The latter could be relevant as higher dosage was required in the OC users, which could make up for less response in OC users relative to non-users. Correspondingly, serum levels of escitalopram seemed to be higher in the OC users, which might also be a result of an OC-induced inhibition of the cytochrome P450 (CYP) hepatic enzymes, CYP2C19 and CYP3A4 (51), which makes up about 70% of the escitalopram metabolism (52).

Preclinical evidence points towards a modulatory role of estradiol on antidepressant drug effects in rats (53–56). One study found that exogenous administration of 17 beta-estradiol as well as ethinylestradiol in ovariectomized female rats facilitates the antidepressant-like effects of fluoxetine and desipramine in a forced swim test (53). In support of that, other studies found that deficiency of brain estrogen levels attenuated sertraline- (54) and duloxetine (55)-induced antidepressive behaviors in mice and it correlated with serotonin turnover in hippocampus and prefrontal cortex (54, 55). In contrast, another study found that acute administration of estrogen with progesterone blocked the antidepressant-like effect of desipramine, but only after acute, not chronic administration (56).

Suppressed levels of endogenous estradiol thus might affect SSRI treatment response and possibly this involves the 5-HT4R. A difference in the 5-HT4R setting could potentially affect the treatment effects as it plays a key role in serotonergic neuronal firing from the dorsal raphe nuclei with projections to most parts of the brain (57).Thus, a lower 5-HT4R agonism capacity could make the serotonergic brain function less adaptable to environmental demands. This aligns with rodent data showing that 5-HT4R partial agonists reduce stress-induced antidepressive behavior (58), have fast acting antidepressant-like effects (59), and that the antidepressant-like effects of SSRIs depends on 5-HT4R activation (60). Whereas our data highlight that treatment response depends on the initial 5-HT4R setting in an OC-dependent manner, we also see a trend towards the expected SSRI-effect on the neostriatal 5-HT4R level after eight weeks of treatment, supporting that the 5-HT4R setting in HC users is sensitive to SSRIs.

## Conclusion and Perspectives

In a depressed cohort, we found no evidence of a difference in the 5-HT4R level between the non-users and the HC users, but the associations between 5-HT4R binding and treatment response were in opposite directions in the OC users and the non-users; the lower the global 5-HT4R binding before treatment start the worse the treatment response in the OC users in contrast to better treatment response in the non-users. We found no strong evidence of a difference in treatment response between the groups. From this study, we speculate that depressed OC users constitutes a special serotonin subtype of MDD which might have implications for SSRI treatment. We will in a planned longitudinal study address the causal relationship between OC use and lower 5-HT4R binding and future and properly-designed studies must address if OC use affects treatment response in depressed women and whether they perhaps could benefit from tapering off OC use. Also, the effects of HIUD use on brain serotonin signatures remains to be investigated in future studies.

### Methodological Considerations

When interpreting the results from this study, some limitations should be considered; 1) the sample size is small, so interpretation should be made with caution, especially with regard to HIUD use and when comparing treatment responses, 2) the OC group was pooled from users of different types of OCs (combined OCs and progesterone-only pills) with different hormone content, which may blur the results, 3) we lacked information on previous HC use among the non-users and information on starting day and pill-free days in the HC users, which could have helped us understand the potential role of HC use in their depressive episode, 4) we had 8 dropouts during follow-up which were mainly OC users. However, based on their last response rate (**Table S2**), they seem more or less equally distributed between those showing some degree of response and those who do not, so it appears not to introduce a selection bias.

## Data Availability Statement

The data analyzed in this study is subject to the following licenses/restrictions: For accessing the dataset, a Cimbi database application is required. Requests to access these datasets should be directed to the corresponding author (vibe.frokjaer@nru.dk) or access can be requested through the procedures outlined here: https://cimbi.dk/index.php/documents/category/3-cimbi-database.

## Ethics Statement

The studies involving human participants were reviewed and approved by The Committees on Health Research Ethics in the Capital Region of Denmark (H-15017713). The patients/participants provided their written informed consent to participate in this study.

## Author Contributions

SL and VF contributed to study conceptualization, data collection, analysis and interpretation of results, participated in drafting the manuscript, and approved the final version prior to submission. BO contributed to analysis and interpretation of data, revised the manuscript critically for important intellectual content, and approved the final version prior to submission. KK-F, AP, VD, and CS contributed to data collection and the acquisition of data, revised the manuscript critically for important intellectual content, and approved the final version prior to submission. GK and MJ contributed to study conceptualization and interpretation of data, revised the manuscript critically for important intellectual content, and approved the final version prior to submission. All authors contributed to the article and approved the submitted version.

## Funding

The study was funded by Independent Research Fund Denmark (grantID: 0134-00278B & DFF-6120-00038), the Research Fund of the Mental Health Services - Capital Region of Denmark, the Innovation Fund Denmark, H. Lundbeck A/S (grantID: 5189-00087A), the Research Council of Rigshospitalet, the Augustinus Foundation (grantID: 16-0058), and Savværksejer Jeppe Juhl og hustru Ovita Juhls Mindelegat. None of the funders had any influence on study design, data collection, analysis, and result interpretation.

## Conflict of Interest

VF and GK declare to have received honorarium for advisory meetings at Sage Therapeutics, GK at Sanos, and VF for a symposium lecture at Lundbeck Pharma A/S. MJ has given talks sponsored by Boehringer Ingelheim and H. Lundbeck.

The remaining authors declare that the research was conducted in the absence of any commercial or financial relationships that could be construed as a potential conflict of interest.

## Publisher’s Note

All claims expressed in this article are solely those of the authors and do not necessarily represent those of their affiliated organizations, or those of the publisher, the editors and the reviewers. Any product that may be evaluated in this article, or claim that may be made by its manufacturer, is not guaranteed or endorsed by the publisher.
