# Supplementary material for: The Impact of Hormonal Contraceptive Use on Serotonergic Neurotransmission and Antidepressant Treatment Response: Results From the NeuroPharm 1 Study
Source: Front Endocrinol (Lausanne). 2022 Mar 11;13:799675. doi: 10.3389/fendo.2022.799675 (PMC8962375; doi:10.3389/fendo.2022.799675)
Supplement: Supplementary file 1 [file DataSheet_1.docx]

Supplementary Material

# Supplementary Tables

**Table S1. Overview hormonal contraception**

| **Type of hormonal contraception** | | **n** | **Ethinylestradiol** | **Gestagen** | **Generation** |
| --- | --- | --- | --- | --- | --- |
| Hormonal intrauterine device | | 11 | - | Levonorgestrel (13.5-20 μg/24 hours) |  |
| Oral contraceptives | | 26 |  |  |  |
|  | Combined oral contraceptives | |  |  |  |
| Femicept | | 4 | 30 μg | Levonorgestrel 150 μg | 2 |
| Malonetta | | 1 | 30 μg | Levonorgestrel 150 μg | 2 |
| Cilest | | 4 | 35 μg | Norgestimat 250 μg | 2 |
| Microgyn | | 4 | 30 μg | Levonorgestrel 150 μg | 2 |
| Microstad | | 1 | 30 μg | Levonorgestrel 150 μg | 2 |
| Daisynelle | | 1 | 20/30 μg | Desogestrel 150 μg | 3 |
| Diane Mite | | 1 | 35 μg | Cyproteronacetat 2 mg | 4 |
| Stefaminelle | | 1 | 20 μg | drospirenon 3 mg | 4 |
| Unknown | | 3 | NA | NA | NA |
|  | Progestogen-only pill |  |  |  |  |
| Cerazette | | 3 | - | Desogestrel 75 μg | 3 |
| Delamonie | | 1 | - | Desogestrel 75 μg | 3 |
| Norethisterone | | 1 | - | Norethisteron 350 μg | 1 |
| Unknown | | 1 | - | NA | NA |

**Table S2. Overview of dropouts**

|  | **Baseline** | | **ΔHAMD6 [%]** | | | | **Escitalopram dose at week 4** | **Switchers to duloxetine** | **Reason for dropout** |
| --- | --- | --- | --- | --- | --- | --- | --- | --- | --- |
| **HC group** | **HAMD6** | **HAMD17** | **Week 1** | **Week 2** | **Week 4** | **Week 8** |  |  |  |
| OC user | 14 | 24 | -7.1 | -7.1 | 14.3 | NA | 10 | Yes | Excluded at week 7 due to adverse side-effects. |
| OC user | 13 | 21 | 0.0 | -7.7 | -15.4 | NA | 15 | No | Drop-out at week 7 due to suicidal attempt. |
| OC user | 14 | 25 | -21.4 | -28.6 | -28.6 | NA | 15 | No | Lost contact at week 7. |
| OC user | 11 | 22 | -90.9 | NA | NA | NA | NA | No | Exclusion at week 1 due to spontaneous remission. |
| OC user | 11 | 20 | -9.1 | -54.5 | -72.7 | -45.5 | 10 | No | Non-compliant to medicine based on blood measures at week 8 |
| OC user | 13 | 25 | -38.5 | -53.8 | NA | NA | NA | No | Lost contact after week 2. |
| Non-user | 17 | 30 | -5.9 | NA | NA | NA | NA | No | Exclusion after week 1, due to hospitalization with suicidal thoughts and psychosis. |
| Non-user | 12 | 24 | -16.7 | -33.3 | -25.0 | -41.7 | 20 | Yes | Non-compliant to medicine based on blood measures at week 8 |
